# *Lasianthus ranongensis* (Rubiaceae), a new species from Andaman tropical rainforest Thailand

**DOI:** 10.7717/peerj.12320

**Published:** 2021-10-12

**Authors:** Tiwtawat Napiroon, Aroon Sinbumroong, Manop Poopath

**Affiliations:** 1 Department of Biotechnology, Faculty of Science and Technology, Thammasat University, Pathum Thani, Thailand; 2 Protected Area Regional Office 4, Department of National Parks Wildlife and Plant Conservation, Surat Thani, Thailand; 3 The Forest Herbarium (BKF), Forest Botany Division, Department of National Parks Wildlife and Plant Conservation, Bangkok, Thailand

**Keywords:** Lasiantheae, Rubiaceae, Morphology, Botany, Tropical rainforests

## Abstract

We describe *Lasianthus ranongensis* Sinbumr. & Napiroon as a new species in the genus *Lasianthus*. The new taxon is intensively discussed through taxonomic affinities and information on its habitat, distribution and conservation status is provided. Moreover, line drawings and stereo microscope images of important fertile organs are demonstrated. The new species is morphologically similar to *L. stipularis* but differs in its having flattened branches (*vs*. terete), leaf blade elliptic-oblong shape of 15.0–20.0 × 4.0–6.0 cm (*vs*. oblanceolate-oblong 12.0–16.0 × 3.0–5.5 cm), 9–12 pairs of veins (*vs*. 9–10 pairs), stipule 5–7 mm long, half covering cymes (*vs*. 10–12.5 mm long, entirely covering cymes), four or five bracts narrowly lanceolate, 2.5–3.0 mm long (*vs*. bracts numerous, broadly triangular, 10–15 mm), flowers with cupular calyx (*vs*. with campanulate calyx), corolla villous on internal surface, and six or seven lobed (*vs*. pubescent on internal surface and four lobed) and drupes with five pyrenes (*vs*. with four pyrenes). It is also similar to *L. pseudo-stipularis*, but from which it is obviously distinguished by its stipule half covers cymes, secondary veins have 9–12 pairs of secondary veins, cupular calyx shape, six or seven lobed corolla, and drupe with five pyrenes, whereas *L. pseudo-stipularis* has stipule entirely covering cymes, 7–8 pairs of secondary veins, obconic calyx, four lobed corolla, and drupe with four pyrenes.

## Introduction

The genus *Lasianthus* Jack (Rubiaceae) includes about 180 species of which a majority of 160 species occur in Asian tropical rainforests (Zhu, Roos & Ridsdale, 2012). In the Southeast Asia, there are more than 142 species which have been treated separately for the different parts of the country (Ridley, 1923; Craib, 1934; Bakhuizen van den, 1965; Zhu, 1998; Zhu, 2001; Naiki et al., 2015). Advances in *Lasianthus* systematics have led to new discoveries, resulting in progress over the last twenty years following the establishment of the tribe Lasiantheae by Bremer & Manen (2000). Such publications have particularly improved our understanding of species distribution and diversity patterns at regional scales. For example, there is a continuous discovery of four new species and nine new recorded in Cambodia (Naiki et al., 2015; Naiki et al., 2017) since the last reports by Pitard (1924). The Malesian region, which includes Malaysia, Indonesia, Brunei, the Philippines, Papua New Guinea, Singapore and the Solomon Islands, 131 species with five subspecies and six varieties were recognized (Zhu, Roos & Ridsdale, 2012). In Thailand, Napiroon et al. (2020) have updated the list and enumerated 68 species based on herbarium specimens and results of their intensive field survey in the natural forests. Based on the synopsis and new species report of the Thai *Lasianthus* species, the genus is divided into two majors groups characterized by shape of cymes: sessile ordominantly pedunculated (Napiroon et al., 2018a; Napiroon et al., 2018b; Napiroon et al., 2018c; Napiroon et al., 2020). Also, presence and absence of bracts and bracteoles are useful characters for classification in *Lasianthus*. This diagnostic significance of peduncles and bracts were emphasized by Hooker (1880), but variation in length of the peduncles sometimes reduces its diagnostic importance. The flowers of *Lasianthus* are fragile and often lack in herbarium specimens thought it is still necessary for identification. Instead, *Lasianthus* could be identified by vegetative characters, such as the shape, size and indumentum on branches, leaf shape, stipules and the calyx (Naiki et al., 2015). Here, we describe a new species from the Andaman tropical rainforest of Thailand by using both completed fertile and vegetative organs collected from our field surveys.

We compared the sample with closely related species, including *L. stipularis* and *L. pseudo-stipularis*, using both completed fertile and vegetative organs. The apparent difference in morphological characters supports its recognition as a new species, which is here described.

## Materials & Methods

### Field surveys and ethics statement

Fieldwork by the Protected Area Regional Office 4, Department of National Parks Wildlife and Plant Conservation was conducted in the Andaman tropical rainforest in Ranong province, located at the West part of southern Thailand in May 2021. They collected an unknown *Lasianthus* species in flowering season from the natural condition. All samples were deposited at the BKF, Department of National Parks Wildlife and Plant conservation for the Flora of Thailand project. The herbarium abbreviations follow the Index Herbarium (Thiers, 2020). Our field studies did not involve any endangered or protected species. Since the species are currently undescribed, they are not currently included in the Threatened Plants of Thailand (Chamchumroon et al., 2017). No specific permits were required for the present study.

### Morphological observation and conservation assessment

We use an exclamation mark (!) to indicate that a type specimen has been seen. Type specimens are cited partly based on online photographs from the herbaria and they are marked as “image!”. Barcodes are given for type specimens together with the relevant literature (Zhu, 1998, 2001, 2002; Zhu, Roos & Ridsdale, 2012). We used the morphological *Lasianthus* species concepts (Zhu, Roos & Ridsdale, 2012; Napiroon et al., 2020) to analyze and compare taxa, placing emphasis on reproductive and vegetative organs such as leaf characters, stipules characters, calyx shape, calyx size, indumentum, corolla characters, the number and morphology of the bracts and the number of pyrenes per drupe. Materials were examined using a stereo microscope (Olympus SZX16, Japan). Terminology and measurements, followed Beentje (2010) and references therein. Conservation assessments for new species follow IUCN Red List categories and criteria (IUCN, 2012; IUCN, 2019).

The electronic version of this article in Portable Document Format (PDF) will represent a published work according to the International Code of Nomenclature for algae, fungi, and plants (ICN), and hence the new names contained in the electronic version are effectively published under that Code from the electronic edition alone. In addition, new names contained in this work which have been issued with identifiers by IPNI will eventually be made available to the Global Names Index. The IPNI LSIDs can be resolved and the associated information viewed through any standard web browser by appending the LSID contained in this publication to the prefix “http://ipni.org/”. The online version of this work is archived and available from the following digital repositories: PeerJ, PubMed Central SCIE, and CLOCKSS.

## Taxonomic results

***Lasianthus ranongensis*** Sinbumr.& Napiroon, sp. nov.

(Figs. 1, 2)

**Figure 1:**
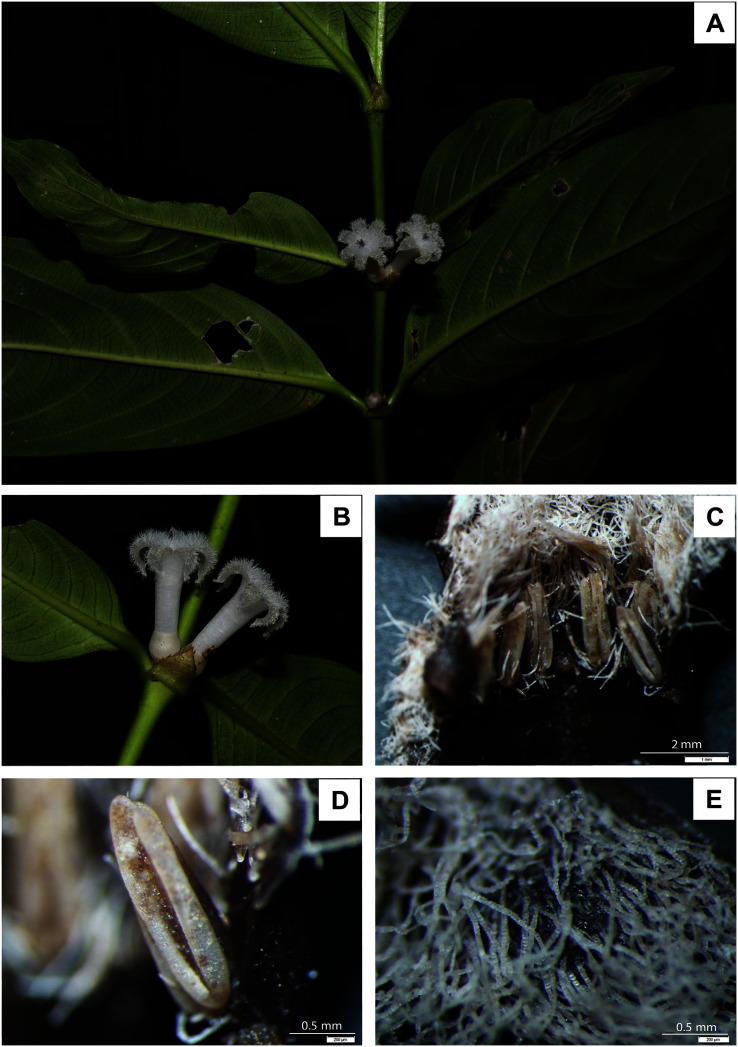
*Lasianthus ranongensis*. (A) Leaf arrangement and branch. (B) Inflorescence and stipule. (C) Six anthers within corolla tube. (D) Anther adnate form. (E) Villous hairs inside corolla. Photos by Mr. Aroon Sinbumroong (A–B), Mr. Niti Panijkasem (C–E).

**Figure 2:**
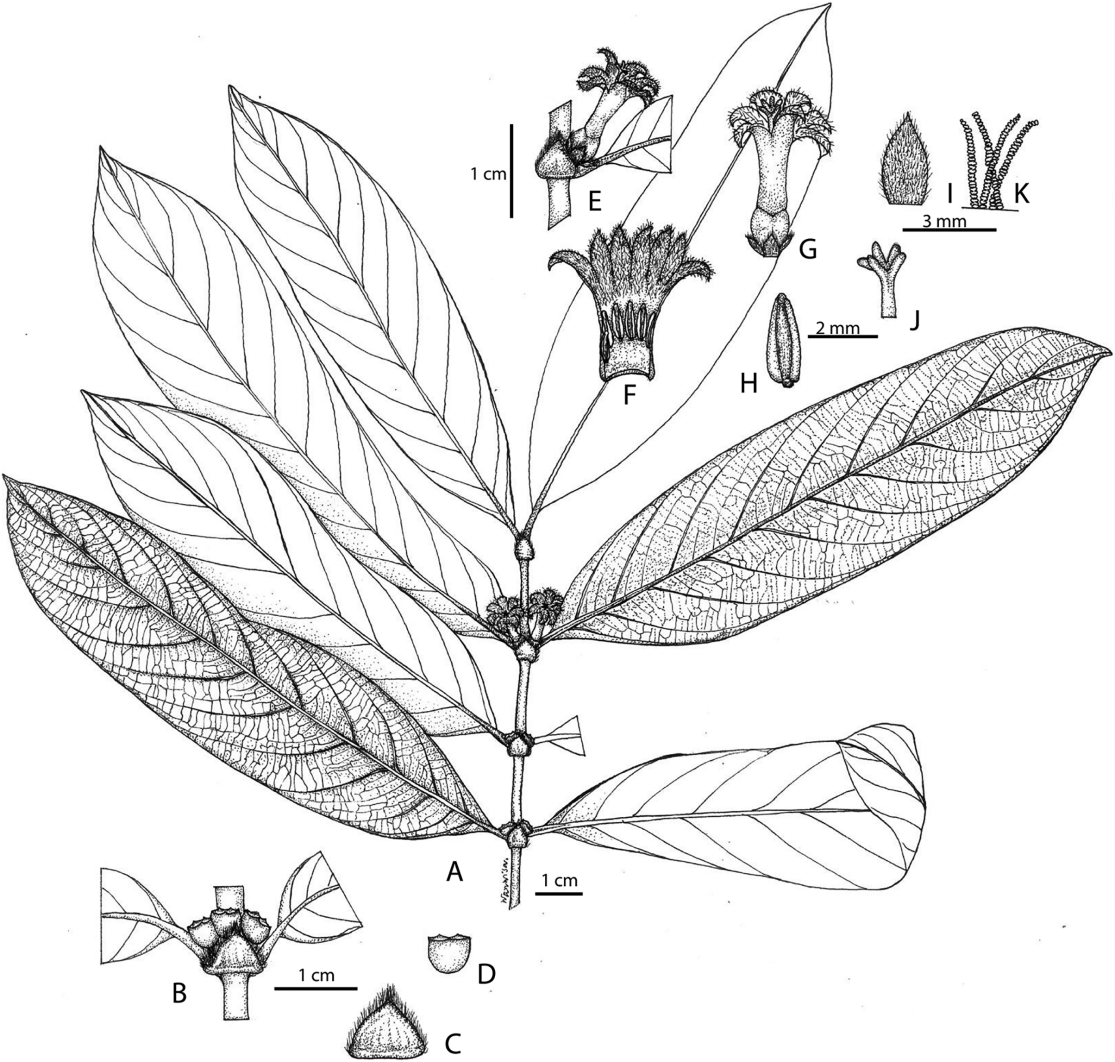
Illustration of *Lasianthus ranongensis*. (A) Leaf shape, Leaf arrangement on branch. (B) Cyme. (C) Stipule. (D) Cupular calyx. (E) Inflorescence. (F) Corolla tube with anthers. (G) Complete flower. (H) Anther adnate, basifixed form. (I) Bract. (J) Stigma with five lobes. (K) Moniliform hairs inside corolla. Illustration by Miss Wanwisa Bhuchaisri.

**Type:–**THAILAND. Ranong Province: Muang Ranong District, Ngao National Park, 10°89′53.2″N, 45°92′88″E, 70 m, 13 May 2021, *A. Sinbumroong 13052021-2* (holotype: BKF!, isotype: BKF!).

LSID to the new species statement: *Lasianthus ranongensis* Sinbumr. & Napiroon: 77220076-1.

**Diagnosis:–***Lasianthus ranongensis* is morphologically similar to *Lasianthus stipularis* Blume, but differs in its having flattened branches (*vs*. terete branches), elliptic-oblong lamina 15.0–20.0 × 4.0–6.0 cm (*vs*. oblanceolate-oblong 12.0–16.0 × 3.0–5.5 cm), 9–12 pairs of secondary veins (*vs*. 9–10 pairs), glabrous young branches (*vs*. puberulous), stipules half covering cymes (*vs*. entirely covering cymes), bracts 4–5, 2.5–3.0 mm long (*vs*. bracts numerous, 10–15 mm long), glabrous cupular calyx, (*vs*. hirsute and campanulate), and 6–7 lobed corolla which inside is villous (*vs*. four-lobed and pubescent inside). In addition, it is similar to *L. pseudo-stipularis* Amshoff ex Bakh.f. but differs by having cupular calyx (*vs*. obconic), six or seven-lobed corolla (*vs*. 4-lobed).

(see Table 1).

**Table 1:** Comparison of diagnostic morphological characters between *Lasianthus ranongensis* and closely related species.

Characters	*L. stipularis* ^1^ ^,^ ^2^ ^,^ ^3^	*L. pseudo-stipularis* ^1^ ^,^ ^4^	*L. ranongensis*
Habit	Shrub 1–2 m high	Shrub 1–2 m high	Shrub, up to three m high
Branches	Terete, 2–3 mm diameter	Terete, 2–3 mm diameter	Flattened, 2–3 mm width
Leaf blade	12.0–16.0 × 3.0–5.5 cm, oblanceolate-oblong	10.0–15.0 × 3.5–5.0 cm, oblong or oblong-obovate	15.0–20.0 × 4.0–6.0 cm, elliptic-oblong
Stipule	10.0–12.5 mm long, broadly triangular-ovate, ovate or ovate-orbicular, entirely covering cymes	5.0–7.0 mm long, triangular-ovate, entirely covering cymes	5.0–7.0 mm long, broadly triangular-ovate, half covering cymes
Secondary veins	9–10 pairs	7–8 pairs	9–12 pairs
Bracts	numerous bracts, narrowly lanceolate 10–15 mm long	bracts minute, inconspicuous or absent	4–5 bracts, narrowly lanceolate 2.5–3.0 mm long
Calyx	calyx hirsute, campanulate, lobes 4, triangular 5–6 mm long	calyx glabrous, obconic, shallowly lobes 4–5, 2 mm long	calyx glabrous, cupular, shallowly lobes 5, five mm long
Corolla	Corolla glabrous outside, pubescent inside, lobes 4, triangular 4–5 mm long	Corolla glabrous outside, pilose inside, lobes 4, triangular 4–5 mm long	Corolla glabrous outside, villous inside, lobes six or seven, triangular five mm long
Drupes	Drupes, ovoid-globose, glabrous	Drupes, globose, glabrous or verrucose	Drupes, globose, glabrous
Number of pyrenes	4	4	5

**Notes:**

1 Zhu, Roos & Ridsdale (2012).

2 Napiroon et al. (2020).

3 Type:—INDONESIA. Java: Salak, *Blume s.n*. (holotype: L! barcode L0000708 image).

4 Type:—INDONESIA. Java: Tjadasmalang, *Winckel 1916ß* (holotype: not seen, isotype: L! barcode U0040464 image).

**Description:–**Shrubs, to three m tall, monoecious. Stem terete, 1.5–2.0 cm in diam., glabrous; branches and branchlets, 2.0–3.0 mm width, flattened, glabrous and glistening. Stipules persistent, broadly triangular-ovate, 5.0–7.0 mm long, apex acute, ciliate. Leaves opposite; petioles flattened, 5–10 mm long, glabrous; blades coriaceous, elliptic-oblong, 15.0–20.0 × 4.0–6.0 cm, base acute, apex short acuminate, glabrous on both surfaces, glistening on adaxial surface, secondary veins 9–12 pairs, slender, slightly depressed above, prominent beneath, ascending at angles of 45°‒50° from midrib, tertiary veins concealed above, obvious beneath, reticulate or netted. Cymes sessile; bracts four to five, narrowly lanceolate, 2.5–3.0 mm long, hirsute at margin. Calyx glabrous and glistering, shallowly five-lobed, tube five mm long; lobes small pointed-like, margin smooth. Corolla narrowly triangular, five mm long, six or seven-lobed; tube 1.0–1.5 mm long, outside glabrous, inside villous; lobes triangular, 5 mm long, externally glabrous, internally villous with moniliform hairs. Stamens 6, anthers, 2.0–2.5 mm long, filament 0.3–0.5 mm long, adnate to corolla. Style three mm long, scabrous, stigma 1.8–2.0 mm long, five-lobed, surface papillate. Drupes globose, young drupes 4–5 mm diam., blue, glabrous; pyrenes 5.

**Common name:–**Dara Pilas, this name refers to the flower which seems like a beautiful star.

**Distribution:–**Endemic to Peninsular Thailand, known from Ngao Sub-district, Muang Ranong District, Ranong Province. It occurs only in tropical rainforest within Ngao National park at the West part peninsular Thailand.

**Habitat:–**Growing in shaded understory near or at the edges of streams in tropical rainforests, on metamorphic or granitic rock substrates at 70 m elevation.

**Proposed IUCN conservation assessment:–***Lasianthus ranongensis* is here assessed as endangered (B2ab(ii)), in accordance with IUCN categories and criteria (IUCN, 2012; IUCN, 2019). It is known from one small population in a protected area at Ngao sub-district, Ranong Province. Its area of occupancy (AOO) is estimated to be less than 500 km^2^. These forests are embraced by agricultural areas and may be devastated by agricultural activities extending nearby. The distribution of these plants is suspected to be declining.

**Phenology:–**Flowering and fruiting in May to June.

**Etymology:–**The specific epithet refers to its occurrence in the Andaman tropical rainforest, Ranong Province, the West part of southern Thailand.

## Discussion

*Lasianthus ranongensis* is a unique species but might be confused with *L*. *stipularis*, which is also found in peninsular Thailand, in having its similar habit and blue colored fruit. However, *Lasianthus ranongensis* is easily set aside from *L. stipularis* by the outstanding characteristics listed in the above diagnosis. In addition, this species is also similar to *L. pseudo-stipularis*. Both have large stipules but *L. ranongensis* presents a stipule with half covering cymes, leaves with 9–12 pairs of lateral veins, and cupular calyx. In contrast, *Lasianthus pseudo-stipularis* has a stipule that entirely covering cymes, leaves with secondary veins 7–8 pairs only, and obconic calyx. In addition, *L. pseudo-stipularis* distributed explicitly in Java and Sumatra in Indonesia.

## Conclusions

This study confirmed that *Lasianthus ranongensis* has morphological evidence as a new species that clearly distinguished from its resemble species. *L. stipularis* and *L. pseudo-stipularis* in both fertile and vegetative characters. These reports are useful for Flora of Thailand project to group of *Lasianthus*.

## Additional Information and Declarations

### Data Availability

The following information was supplied regarding data availability: The raw data is available in the Table.

### New Species Registration

The following information was supplied regarding the registration of a newly described species: LSID to the new species statement: *Lasianthus ranongensis* Sinbumr. & Napiroon: 77220076-1.
